# Spatiotemporal Pattern of Urban-Rural Integration Development and Its Driving Mechanism Analysis in Hangzhou Bay Urban Agglomeration

**DOI:** 10.3390/ijerph19148390

**Published:** 2022-07-09

**Authors:** Caiyao Xu, Chen Qian, Wencai Yang, Bowei Li, Lingqian Kong, Fanbin Kong

**Affiliations:** 1 Institute of Ecological Civilization, Zhejiang A&F University, Hangzhou 311300, China; xucaiyao@zafu.edu.cn (C.X.); lbw@zafu.edu.cn (B.L.); arvin-kong@zafu.edu.cn (L.K.); 2 Research Academy for Rural Revitalization of Zhejiang Province, Zhejiang A&F University, Hangzhou 311300, China; 3 College of Economics and Management, Zhejiang A&F University, Hangzhou 311300, China; 2021606022057@stu.zafu.edu.cn (C.Q.); 2020106011019@stu.zafu.edu.cn (W.Y.)

**Keywords:** urbanization, urban agglomeration, geographical detector model, Hangzhou Bay

## Abstract

The quantitative analysis of the urban-rural integration development (URID) level and its driving factors is of great significance for the new-type urbanization of urban agglomerations. This study constructed a multidimensional framework in the perspective of a population–space–economy–society–ecology framework to measure the URID level from 2000 to 2020 and further explored the driving mechanism of the URID changes by a geographical detector model in the Hangzhou Bay urban agglomeration (HBUA). The results showed that the land-use change in the HBUA from 2000 to 2020 showed a typical characteristic of the transition between cultivated and construction land. The URID level in the HBUA improved from 0.294 in 2000 to 0.563 in 2020, and the year 2005 may have been the inflection point of URID in the HBUA. The URID level showed a significant spatial aggregation with high values. Hangzhou, Jiaxing, and Ningbo were hot spots since 2015, and the cold spots were Huzhou and Shaoxing. The population and spatial integration had more important impacts on URID levels in 2000, 2005, and 2020, while economic and social integration had more significant impacts on URID levels in 2010 and 2015. This study provided a deeper understanding of the evolution of URID in an urban agglomeration and could be used as a reference for decision makers.

## 1. Introduction

Urbanization and industrialization play important roles in the urban-rural relationship and have significantly widened the gap between urban and rural areas. Rural decline, such as depopulation and poverty, has become a common phenomenon in many countries around the world [1,2,3,4,5]. Therefore, the contrast between rural decline and urban prosperity has been obvious, causing countries to revitalize their countryside [6]. For example, the European Union’s common agricultural policy, launched in 1962, aimed to support farmers, improve agricultural productivity, and keep the rural economy alive, as well as foster rural development in terms of ecosystems and sustainable management [7]. The concept of urban-rural equalized development was proposed in Germany in 1950 and stated that “inhabitants in rural and urban areas may have different lifestyles, but enjoy an equivalent standard of living” [8]. Moreover, the urban-rural interactions and urban-rural teleconnections have been key themes in the Global Land Programme, which aimed to understand the feedback between urban and rural environments [9]. Many research fields have been driven and fostered to build better relationships and development between urban and rural areas [10,11,12]. China, with the largest population in the world, still has a dual economy, namely, an economically advanced sector and an economically underdeveloped sector, which was revealed by the British economist W. Arthur Lewis in 1954. The ratio of urban to rural income was 1.86:1 in 1985, it peaked in 2009 at 3.33:1 [2], and was 2.39 in the first quarter of 2022 [13]. The disparities between rural and urban areas in China have been obvious and significant, including housing [14], infrastructure [15], environment management [16], planning [17], social services [18], and quality of life [19]. Therefore, narrowing the gap between urban and rural areas is an urgent problem in China.

Urban-rural development in a megacity or urban agglomeration has been the focus as it indicates the future development of the whole country [2]. URID is the solution to the disparities between rural and urban areas and has been a priority task in China [20]. It has also been beneficial to high-quality development and the goal of common prosperity [21,22]. The government at each level in China has released a related policy to foster the development of urban-rural integration. China’s national government issued the “Opinions on Establishing and Improving the Institutional Mechanism and Policy System for Urban-rural Integrated Development” in 2019. The “Outline of the 14th Five-Year Plan (2021–2025) for National Economic and Social Development and Vision 2035 of the People’s Republic of China” was released in 2021 and further indicated that it is necessary to “strengthen the systems and mechanisms of integrated urban-rural development” [23]. The “Key Tasks for a New-type of Urbanization and Urban-rural Integration Development in 2021” specifically indicated that it was necessary to change the development mode of megacities and accelerate the promotion of urban-rural integration development [24]. The “Key Tasks for a New-type of Urbanization and Urban-rural Integration Development in 2022” were to improve the integrated development mechanism of urban agglomerations [25]. Moreover, 11 areas were selected to develop as national pilot zones for urban-rural integrated development [26]. Creating a new pattern of high-quality development in urban-rural integration is a major strategic need in China to promote the coordinated and sustainable development of urban and rural societies, economies, and environments so as to meet the growing needs of the people for a better life. Therefore, it is urgent to understand the changing tendency of urban-rural integration development and its driving mechanism, which can provide reference for the sustainable planning and decision-making in China and other developing countries.

Previous research has mainly focused on the characteristic of URID. The quantitative measurement of the URID level is a key first step. A basis–driver–goal framework was built and applied to make the evaluation index system, which further measured the regional differentiation and explored the spatiotemporal evolution of the URID levels in China from 2000 to 2018 [27]. Another multidimensional evaluation index system based on the people–space–economy–society–ecology for urban-rural integration analyzed the URID levels in China from 1999 to 2016 [28]. Moreover, researchers quantitatively analyzed the evolution rules and spatiotemporal patterns of land-use transitions and URID in China, which revealed that land-use transitions negatively impacted URID [29]. Furthermore, studies also focused on the difference in URID from different perspectives, such as quality of life [19] and equity [30]. These studies provide a helpful basis to understand the URID, but the spatiotemporal variation and driving mechanism of URID in developed urban agglomerations have not yet been fully explored.

The HBUA is located in the Yangtze River Delta region, which is the most dynamic and economically developed area after China’s reform and opening. Furthermore, the HBUA is an optimized development area in the “Outline of integrated development of Yangtze River Delta” [31] and includes one of the 11 national pilot zones for the URID. Therefore, how to take the lead in optimizing the development model and build a new pattern of the URID in this region has a typical demonstration and is of great significance for promoting the integration development of the Yangtze River Delta. Therefore, the objectives of this study were to analyze the characteristic of urbanization according to land-use change, quantitatively measure the URID level, explore the spatiotemporal pattern of URID, and further reveal the driving mechanism of the variation in URID levels. Finally, some suggestions for promoting the URID were discussed.

The rest of this paper is organized as follows. Section 2 is a theoretical analysis that describes the connotation of URID as well as the literature on the measurement of URID in the context of China, which provides a reference for the measurement indicator system of URID in Section 3. Section 3 discusses the methodology as well as the data source. Section 4 presents our results, and Section 5 discusses the significance of our findings as well as the implications and limitations. Section 6 presents the conclusions of this study.

## 2. Theoretical Analysis and Hypothesis

### 2.1. Theoretical Framework

**Figure 1:**
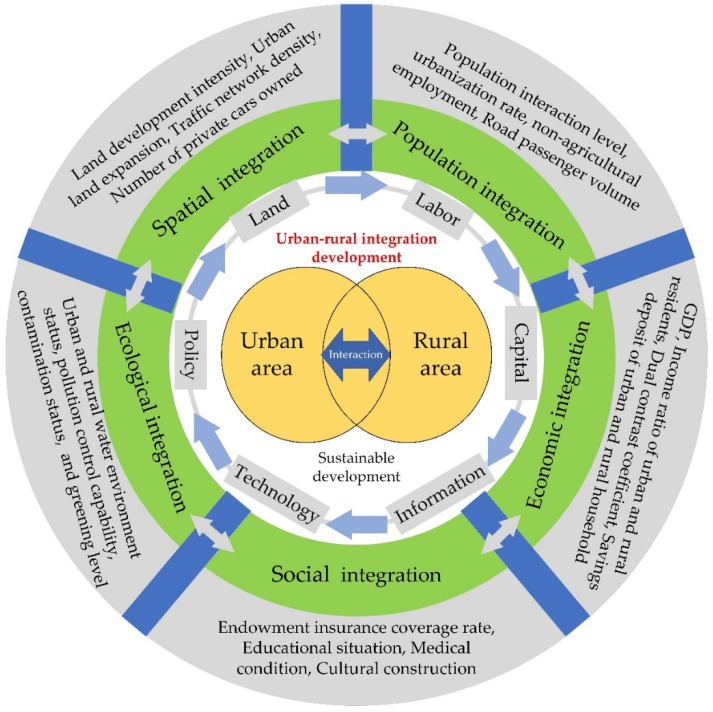
The theoretical framework of URID. GDP represents the gross domestic product.

URID was intended to solve the problem of insufficient and unbalanced development during urbanization, that is, to pursue an equalized development between urban and rural areas [8]. The policies of new-type urbanization and rural revitalization in China [32] aimed to make the society more sustainable and ultimately achieve common prosperity. Therefore, the process of urban-rural integration should close the urban-rural divide in order to achieve equity, where urban and rural areas are regarded as a single area, foster the sufficient and balanced flow of the diverse resources and elements between urban and rural areas, and build an effective urban-rural connection through the integration of the economy, the society, and the ecological environment (Figure 1).

The strategy of people-centered urbanization confirmed that the target of URID was to meet the growing needs of the people for a better life [25]. Talent has been a key factor in the integrated development of urban and rural areas. Off-farm employment for rural labor has been driven by urban growth and has significantly contributed to rural development [33]. Furthermore, migrant workers impact the economic, social, and cultural conditions in the urban areas [34]. It is necessary to fully revitalize the human resources in urban and rural areas and ensure a bidirectional flow of talent for the benefit of both areas. Urban-rural integration should promote a society toward equality, rather than artificial social stratification. Therefore, the immigrant and the emigrant population, the proportion of the urban population, and the proportion of employees in the secondary and tertiary industries should be benchmarks for the population integration between urban and rural areas.

Continuously optimizing the spatial layout and pattern of urbanization and rural construction can ensure land for equal and orderly development. Research demonstrates that land-use transition plays a negative role in the URID in China from 2005 to 2016 [29]. Furthermore, as an important land-use type, roads can provide access to local and regional job markets for migrant farmers and they have a positive influence on narrowing the income gap between urban and rural areas [35] and economic development [36]. Therefore, the land development intensity, urban land expansion, traffic network density, and the number of private cars owned could be used to describe the spatial integration between urban and rural areas.

The main body of URID contains equalized social services and sufficient cooperation in economic development. Social services, such as healthcare, education, and recreational infrastructure have high heterogeneity between urban and rural areas [18,37]. The migrants from rural to urban areas have resulted in school disruptions for children [38], vulnerability to social exclusion [39], and other social issues. This has been attributed to fewer employment opportunities and the lack of public service facilities in urban fringe and rural areas, which play negative roles in quality of life [40]. Moreover, the wealth and income gaps between urban and rural areas have been significant due to the heterogeneous social services available [41], as well as the impact of income level on mental health [42]. Studies have found that building a more civilized and harmonious society is beneficial in terms of sustainable development [43]. Therefore, the endowment insurance coverage rate, educational situations, medical condition, and cultural construction could be used to describe the social integration between urban and rural areas. The GDP, the income ratio of urban and rural residents, the dual contrast coefficients, and the financial stability of urban and rural households could be selected to describe the economic integration between urban and rural areas.

Lastly, constraining development activities within the capacity of the resources and the environment is important for sustainability. Urbanization commonly has damaged the ecological environment [44]. The typical surface urban heat island, the household waste, and the non-point-source pollution from agriculture have been confirmed in the process of urban-rural transition and integration [45,46,47]. Therefore, the urban and rural water environment status, pollution control capability, contamination status, and greening level could be used to describe the ecological integration between urban and rural areas.

Overall, the URID is a long-term evolution view, which was based on the preservation of the basic characteristics of urban and rural areas and driven by new-type urbanization and rural revitalization strategies to promote the integrated development of urban and rural areas in terms of people, space, economy, society, and ecology, and ultimately achieve common prosperity [25]. Therefore, the URID represents the interaction and integration of population, space, economy, society, and ecology between urban and rural areas (Figure 1).

### 2.2. Hypothesis

The URID in China has been promoted during the process of rapid urbanization and industrialization, especially after their reform and opening. As for the URID level, research has demonstrated that the overall URID level in China from 2000 to 2018 exhibited a U-shaped curve, with 2006 as the inflection point that was characterized by significant spatial agglomeration [27]. Another study in China from 2005 to 2016 indicated that the URID level had improved since 2005 and was characterized by spatial agglomeration [29]. Through the above analysis, three hypotheses were proposed, as follows:

**Hypothesis** **1.** *The URID level of the HBUA has improved from 2000 to 2020.*

**Hypothesis** **2.** *The year 2005 may be the inflection point of the URID evolution of the HBUA.*

**Hypothesis** **3.** *The URID level in the HBUA has been characterized by significant spatial agglomeration*.

The URID should be driven by environmental conditions and socio-economic factors. In the Pearl River Delta Region, traffic location was the most significant factor in the urban-rural spatial transformation process [48]. Land-use transition had a negative impact on URID in China [29]. Other research revealed that policies and institutional structures, economic growth, and urbanization were the driving factors [8]. In the five-dimension framework (Figure 1), the URID was influenced by various factors. Through the above analysis, another hypothesis was proposed.

**Hypothesis** **4**. *The driving factor of the URID evolution in the HBUA was different at different stages of development*.

## 3. Materials and Methods

### 3.1. Study Area

**Figure 2:**
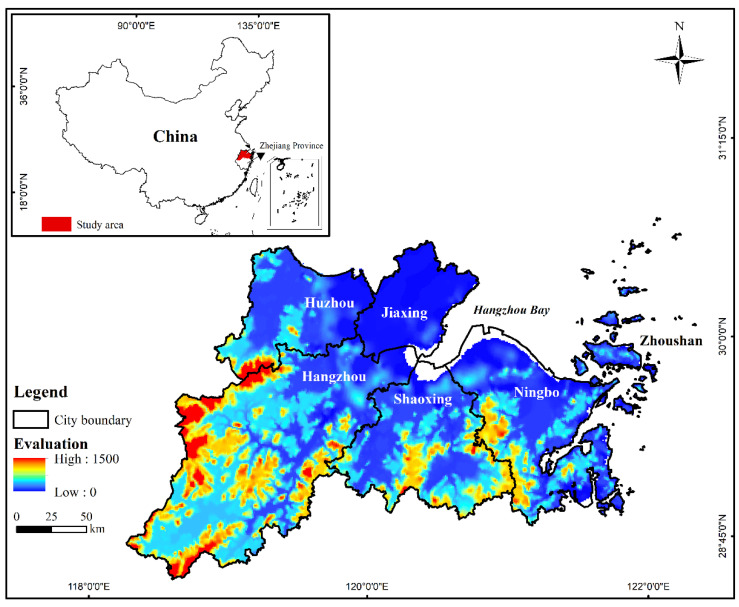
The location of the study area.

The study area, the Hangzhou Bay urban agglomeration (HBUA), is located in Zhejiang Province (Figure 2) and includes six megacities (Hangzhou, Huzhou, Jiaxing, Ningbo, Shaoxing, and Zhoushan), two (Jiaxing and Huzhou) of which are the national pilot zones for urban-rural integrated development in China [49]. The HBUA has a total population of approximately 36.6 million, a total GDP of CYN 4473.9 billion, and an area of 46,447 km^2^ as of 2020 [50]. Hangzhou and Ningbo have a total GDP of more than CYN 1 trillion in 2020. The Outline of the 14th Five-Year Plan (2021–2025) for the National Economic and Social Development and Vision 2035 of the Zhejiang Province [51] was released in 2021 and promoted the construction of Hangzhou Bay as a large, modernized bay and the integrated development of urban and rural areas in Zhejiang Province. The plans for Hangzhou Bay aimed to strengthen its core, leading position of economic development in Zhejiang Province [52], such as “The Industrial Zone Development Plan of HBUA” (released in 2003) [53], “Strategic Planning for Spatial Development of HBUA (2004–2020)” (released in 2003) [54], and “Development Plan for High-tech Industrial Belt around HBUA” (released in 2020) [55], which made the HBUA a typical area to explore the spatiotemporal pattern of urban-rural integration development and its driving mechanisms.

### 3.2. Data Collection and Processing

Land-use data from 2000 to 2020 was acquired from the Resource and Environment Science and Data Center (https://www.resdc.cn/ (accessed on 4 July 2022). Socio-economic data from 2000 to 2020 were extracted from the statistical yearbook of the six cities (Hangzhou, Huzhou, Jiaxing, Ningbo, Shaoxing, and Zhoushan) and Zhejiang Province [50,56,57,58,59].

### 3.3. Methodology

#### 3.3.1. Measurement of the Urban-rural Integration Development

The indicator system for measuring the level of URID was constructed based on the theoretical analysis in Section 2 and the related research [27,28,60,61], including five first-level indicators such as population integration, economic integration, social integration, spatial integration, ecological integration, and 20 second-level indicators (Table 1). The data source was at the city level, and the study periods included 2000, 2005, 2010, 2015, and 2020; so, the total number of each variable sample was 30.

The urban–rural integration development index (URIDI) was built by using the entropy weight method [62] and Equation (3) after the standardization of indicators to quantitatively analyze the level of URID of the HBUA in 2005, 2010, 2015, and 2020.

The standardization of indicators is as follows:

(1) $$R_{i}=\frac{\mathrm{Xi}-X_{\min}}{X_{\max}-X_{\min}}\;(\mathrm{Positive}\;\mathrm{indicator})$$

(2) $$R_{i}=\frac{X_{\max}-\mathrm{Xi}}{X_{\max}-X_{\min}}\;(\mathrm{Negative}\;\mathrm{indicator})$$

where R_i_ is the standardized value of indicator i, Xi is the actual value of indicator i in Table 1 (i = 1~20), X_max_ is the maximum value of indicator i, and X_min_ is the minimum value of the indicator i.

The calculation of the URIDI is as follows:

(3) $$\mathrm{URIDI}=w_{1}X1+w_{2}X2+w_{3}X3+\ldots +w_{i}\mathrm{Xi}$$

where w_i_ is the weight of indicator i.

#### 3.3.2. Hotspot Analysis

Based on the ArcGIS 10.5 software platform, a 5 km × 5 km grid was created, and a total of 1681 grid points were obtained to extract the URIDI values. Hotspot analysis (Getis-Ord Gi*) was applied to explore the spatial pattern of the URIDI values of the HBUA in 2000, 2005, 2010, 2015 and 2020. The equation of Gi* is given as [63]:

(4) $${\mathrm{Gi}}^{*}=\frac{\sum_{j=1}^{n}w_{i,j}x_{j}-\;\bar{x}\sum_{j=1}^{n}w_{i,j}}{s\sqrt{\frac{n\sum_{j=1}^{n}w_{i,j}^{2}-{\left(\sum_{j=1}^{n}w_{i,j}\right)}^{2}}{n-1}}}$$

(5) $$s=\sqrt{\frac{\sum_{j=1}^{n}x_{j}^{2}}{n}-{\left(\;\bar{x}\right)}^{2}}$$

(6) $$\;\bar{x}=\frac{\sum_{j=1}^{n}x_{j}}{n}$$

where Gi* is a z-score, x_j_ is the attribute value of feature j, w_i,j_ is the spatial weight between feature i and feature j, n is the total number of features, and s is the standard deviation.

**Figure 3:**
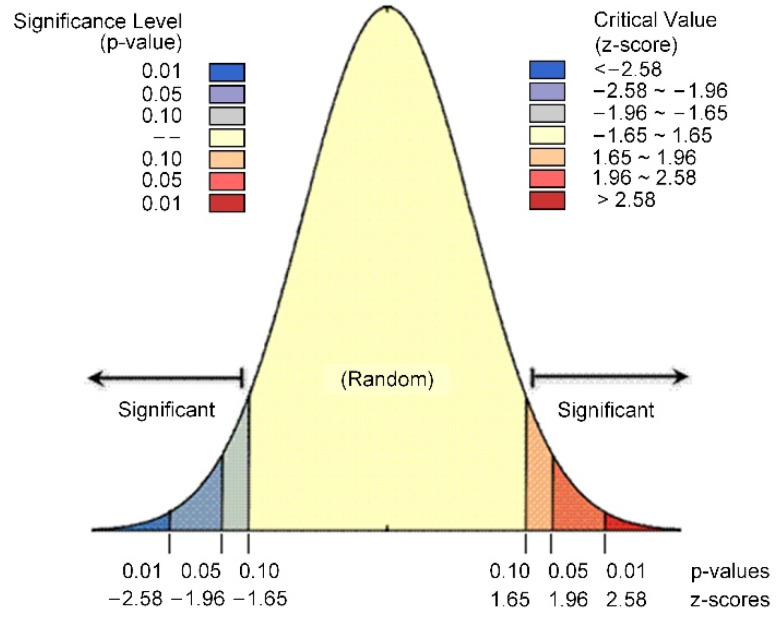
The result of statistical significance testing for Gi*.

The result of statistical significance testing for Gi* is shown in Table 2 and Figure 3.

#### 3.3.3. Geographical Detector Model

The geographical detector model is a statistical tool to measure regional spatial stratified heterogeneity that was built by Jinfeng Wang et al. [64] and further applied in many kinds of research [65,66,67,68,69]. The geographical detector model includes a factor detector, interaction detector, risk detector, and ecological detector.

The factor detector contains the detection of spatial differentiation of Y; the detection of variable X explains the spatial differentiation of Y. The result of the factor detector revealed the relative importance of explanatory variables with a Q-statistic [64,70,71].

(7) $$Q=1-\frac{\sum_{h=1}^{L}N_{h}\sigma_{h}^{2}}{{N\sigma }^{2}}=1-\frac{\mathrm{SSW}}{\mathrm{SST}}$$

(8) $$\mathrm{SSW}=\overset{L}{\underset{h=1}{\sum }}N_{h}\sigma_{h}^{2}$$

(9) $$\mathrm{SST}={N\sigma }^{2}$$

where h is the strata of Y (i.e., URIDI) or variables X, h = 1, …, L. N_h_ and N represent the number of units in the strata h and the whole study area, respectively. The $\sigma_{h}^{2}$ and $\sigma^{2}$ are the variance of Y (i.e., URIDI) in the strata h and the whole study area, respectively. SSW and SST are within the sum of squares and the total sum of squares, respectively. The Q value, which is between 0 and 1, indicates that X explains 100 × Q% of Y. If the Q value of variable X is 1, then X completely controls the spatial distribution of Y; if the Q value is 0, then X has no relationship with Y.

The interaction detector determines the interactive impacts of two overlapped spatial variables based on the relative importance of interactions computed with Q values of the factor detector [64,70,71]. The types of the interaction detector are shown in Table 3.

The ecological detector is used to compare whether the influence of two explanatory variables, X1 and X2, on the spatial distribution of Y (i.e., URIDI) is significantly different, which is measured by F statistic [64,70,71].

(10) $$F=\frac{N_{X1}\left(N_{X2}-1\right){\mathrm{SSW}}_{X1}}{N_{X2}\left(N_{X1}-1\right){\mathrm{SSW}}_{X2}}$$

(11) $${\mathrm{SSW}}_{X1}=\overset{L1}{\underset{h=1}{\sum }}N_{h}\sigma_{h}^{2}$$

(12) $${\mathrm{SSW}}_{X2}=\overset{L2}{\underset{h=1}{\sum }}N_{h}\sigma_{h}^{2}$$

where N_X1_ and N_X2_ represent the number of samples of variables X1 and X2, respectively. SSW_X1_ and SSW_X2_ are within the sum of the squares of the variables X1 and X2, respectively. L1 and L2 are the number of strata of X1 and X2, respectively.

The package GD in R (version 4.1.2, R Development Core Team) was applied to implement the geographical detector model and draw the figures [71].

## 4. Results

### 4.1. Characteristics of Urbanization Based on Land-Use Change

**Figure 4:**
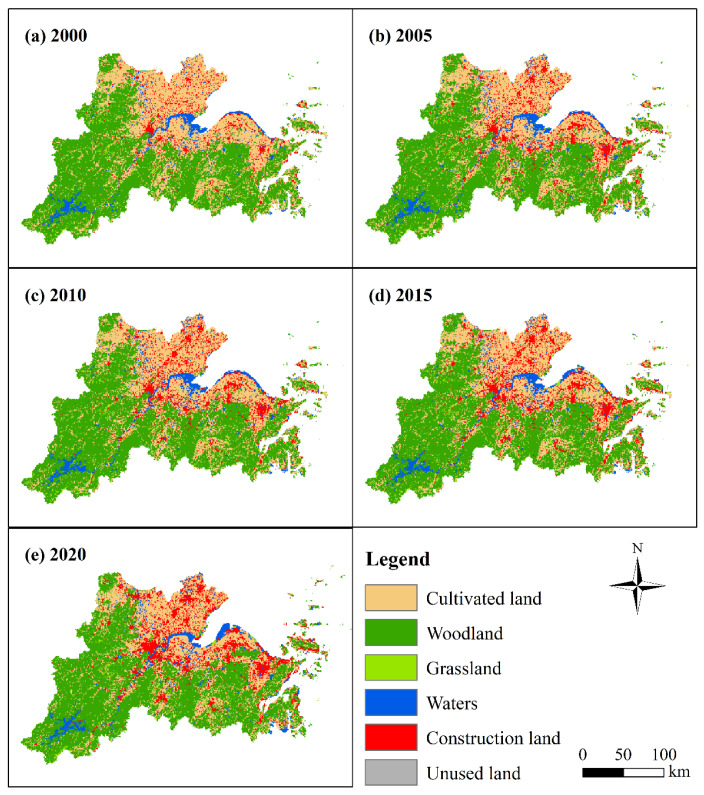
Characteristics of land-use change in HBUA from 2000 to 2020.

**Figure 5:**
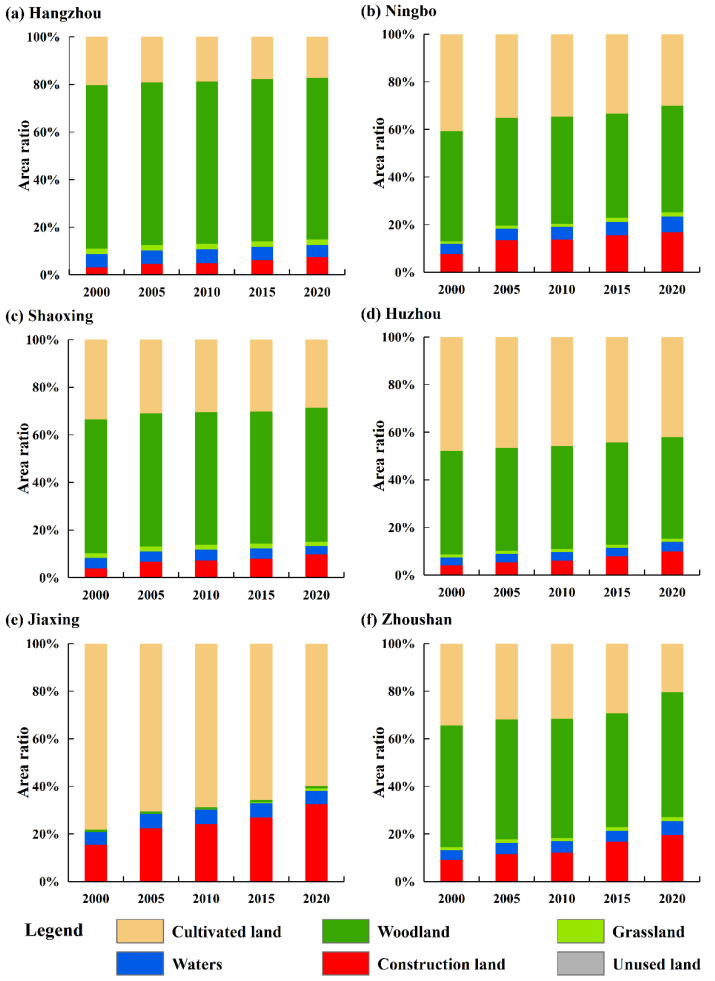
Area proportion of different land-use types in HBUA from 2000 to 2020.

The characteristics of land-use change and the area ratio of each land-use type at the city-level scale in the HBUA from 2000 to 2020 are depicted in Figure 4 and Figure 5, respectively, showing the characteristics of the continuous encroachment of cultivated land by construction land. Specifically, the area of cultivated land decreased from 40.774% in 2000 to 30.844% in 2020, while the area of construction land increased from 8.081% in 2000 to 18.092% in 2020. The other land-use types, such as woodland, water area, grassland, and unused land, were relatively stable, and their area proportions remained at 38.2%, 11.3%, 1.4%, and 0.1%, respectively.

At a city-level scale, the main land-use type in Hangzhou was woodland, and its area proportion remained at approximately 68% from 2000 to 2020. The area proportion of cultivated land decreased from 20.368% in 2000 to 17.192% in 2020, and the area of construction land increased from 3.177% in 2000 to 7.448% in 2020 (Figure 5a). The main land-use type in Ningbo was woodland, and its area proportion remained at about 45% from 2000 to 2020. The area ratio of cultivated land decreased from 40.773% in 2000 to 29.955% in 2020, and the area of construction land rose from 7.719% in 2000 to 16.683% in 2020 (Figure 5b). The main land-use type in Shaoxing was woodland, and its area proportion remained at about 56% from 2000 to 2020. The area proportion of cultivated land decreased from 33.410% in 2000 to 28.573% in 2020, and the area ratio of construction land increased from 3.873% in 2000 to 9.852% in 2020 (Figure 5c). The land-use type in Huzhou was dominated by cultivated land and forest land. The area proportion of forest land remained at approximately 43%, and the proportion of cultivated land area decreased from 47.786% in 2000 to 42.103% in 2020. The area of construction land increased from 4.119% in 2000 to 10% in 2020 (Figure 5d). Cultivated land was the main land-use type in Jiaxing, and its area proportion decreased from 78.252% in 2000 to 59.902% in 2020. The proportion of construction land in Jiaxing increased from 15.513% in 2000 to 32.479% in 2020, and the proportion of woodland was approximately 1%. The main land-use type in Zhoushan was woodland, and its area ratio fluctuated from 47.993% (2015) to 52.513% (2020). The proportion of cultivated land decreased from 34.390% in 2000 to 20.436% in 2020, and the proportion of construction land increased from 9.040% in 2000 to 19.514% in 2020.

### 4.2. Temporal Characteristics of Urban-rural Integration Development Level

**Figure 6:**
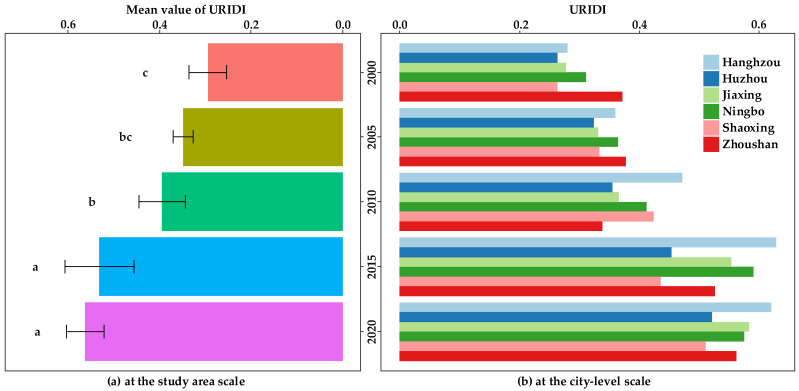
Temporal characteristics of URID level at study-area scale (**a**) and city-level scale (**b**). In (**a**), the same lowercase letter indicates that the URIDI has no significant difference between different years (*p* > 0.05) and different lowercase letters indicate that the URIDI has a significant difference between different years (*p* < 0.05). The error bars represent the standard deviation.

The overall URID level of the HBUA showed a growing trend from 2000 to 2020 (Figure 6a), increasing from 0.294 in 2000 to 0.563 in 2020. There was a significant difference among 2000, 2010, and 2015 (*p* < 0.05); there was no significant difference between 2015 and 2020 (*p* > 0.05). From the perspective of the city-level area (Figure 6b), Hangzhou’s URIDI value increased from 0.280 in 2000 to 0.621 in 2020, with an increase of 121.98%. The URIDI value of Huzhou increased from 0.264 in 2000 to 0.522 in 2020, with an increase of 97.81%. The URIDI value of Jiaxing increased from 0.277 in 2000 to 0.584 in 2020, with an increase of 110.71%. Ningbo’s URIDI value increased from 0.311 in 2000 to 0.576 in 2020, with an increase of 85.11%. The URIDI value of Shaoxing increased from 0.263 in 2000 to 0.511 in 2020, with an increase of 94.13%. Zhoushan’s URIDI value increased from 0.372 in 2000 to 0.562 in 2020, with an increase of 51.26%. Among the URIDI values of these six cities, Hangzhou has ranked first since 2010.

### 4.3. Spatial Pattern of Urban-Rural Integration Development Level

**Figure 7:**
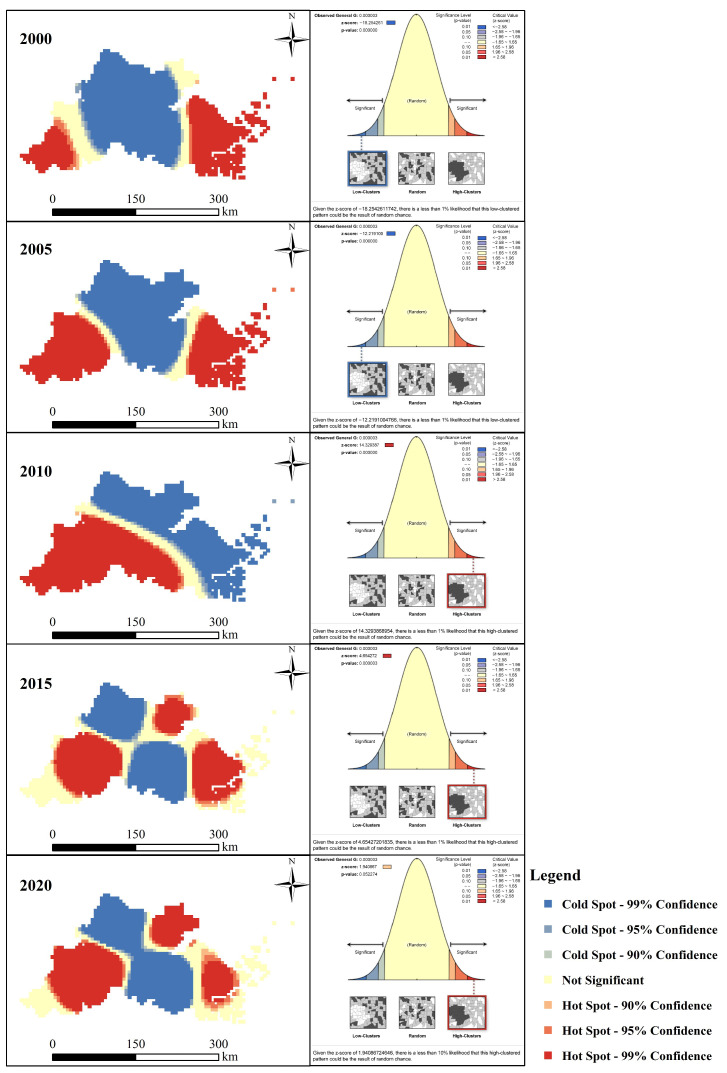
Spatial pattern characteristics of URID in HBUA from 2000 to 2020.

The results of the spatial pattern characteristics of the urban-rural integration development level of the HBUA in 2000, 2005, 2010, 2015, and 2020 are shown in Figure 7. The URID level of the HBUA showed an obvious spatial aggregation. The URID level in 2000 and 2005 showed the characteristics of significant low-value clustering (*p* < 0.01). Low values were concentrated in Huzhou, Shaoxing, and Jiaxing. The URID level in 2010 (*p* < 0.01), 2015 (*p* < 0.01), and 2020 (*p* < 0.1) showed the characteristics of significant high-value clustering. Among them, in 2010, it showed the characteristics of “low-value aggregation in the northeast and high-value aggregation in the southwest”, which mainly showed that the high value was concentrated in Hangzhou and Shaoxing and the low value was concentrated in Jiaxing, Zhoushan, Huzhou, and Ningbo. In 2015 and 2020, the low-value agglomeration areas were Shaoxing and Huzhou and the high-value agglomeration areas were Hangzhou, Jiaxing, and Ningbo.

### 4.4. Driving Mechanism of Urban-Rural Integration Development-Level Change

**Figure 8:**
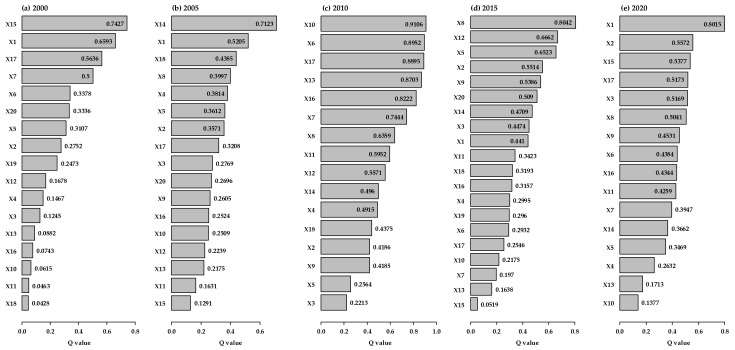
Contributions of variables to URID changes investigated by the factor detector from 2000 to 2020.

The Q values and ranks of variables investigated by the factor detector from 2000 to 2020 are described in Figure 8. The main driving factors of urban-rural integration development in the HBUA varied in different years. The indicators of population integration (X1) and spatial integration (X13, X14, and X15) had more important impacts on the URID level in 2000, 2005, and 2020, while economic integration (X8, X6, X5) and social integration (X10, X12) had more significant impacts on the URID level in 2010 and 2020. In addition, the Q value of X1 decreased from 0.6593 in 2000 to 0.5205 in 2005 and increased to 0.8015 in 2020.

**Figure 9:**
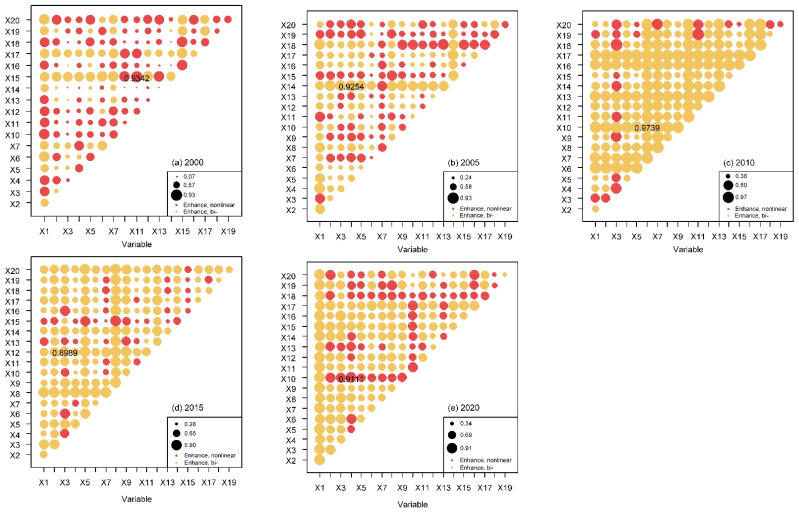
Interaction detection of influencing factors of URID level in HBUA from 2000 to 2020. The interaction with the highest Q value in each year is marked. The size of the dots represents the Q value of the interaction between two variables. The different colors indicate the interaction types of each two variables (Table 3). The variables X8 and X9 in 2000 were excluded because their variances both were 0.

**Figure 10:**
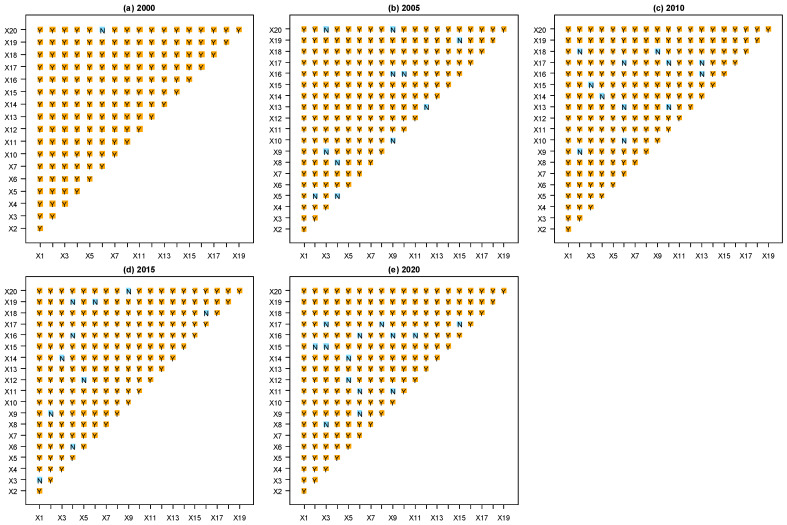
The ecological detector result of influencing factors of URID level of HBUA from 2000 to 2020. “Y” represents a statistically significant difference between the two variables with the confidence of 95%; “N” indicates that the two variables had no significant difference. The variables X8 and X9 in 2000 were excluded because their variances both were 0.

The interactions between variables on URID in the HBUA from 2000 to 2020 were explored by the interaction detector (Figure 9) and further analyzed by using the F-statistic of the ecological detector (Figure 10) to reveal the significance of the different influence of variables. Overall, the interaction values of the two factors were greater than the maximum value of a single factor (Figure 8), indicating that the impact of each variable on URID was not independent but synergistically enhanced. The interaction with the highest Q value (0.9342) in 2000 was between X11 and X15 (*p* < 0.05), and the interaction values between X15 and X13, X7, X12, X17, X10, and X18 were more than 0.87 (*p* < 0.05). In 2005, the interaction with the highest Q value (0.9254) was between X4 and X14 (*p* < 0.05), and the interaction values between X14 and X3, X2, X5, X17, and X9 were more than 0.85 (*p* < 0.05). In 2010, the Q values of X6 and X10 were the largest (0.974, *p* < 0.05), and the interactions between X6 and X17, X11, X3, X7, and X20 were also strong (*p* < 0.05), the Q value of which were more than 0.95. In 2015, the interaction with the highest Q value (0.8989) was between X3 and X12 (*p* < 0.05). There were also strong interactions between X8 and X20, X12, X19, X5, X17, and X2, the Q values of which were more than 0.87 (*p* < 0.05). In 2020, the interaction value between X4 and X10 was the largest (0.9111, *p* < 0.05) and there were strong interactions between X1, and X19, X3, X10, X20, X5, X2, and X16 (*p* < 0.05), the Q values of which were more than 0.86.

## 5. Discussion

### 5.1. Characteristics of Urban-Rural Integration Development in Urban Agglomeration

A population–space–economy–society–ecology framework was constructed in this study to depict the URID and measure it with the URIDI. The URID level in the HBUA improved from 2000 (the URIDI value was 0.294) to 2020 (the URIDI value was 0.563). This result validated Hypothesis 1. Considering the significant differences among 2000, 2010, and 2015, 2005 could have been the inflection points of URID, similar to the results of studies on the URID level in China [27,29]. This result validated Hypothesis 2. According to the classification and discrimination criteria of the URID level in a previous study [72], in 2020, Hangzhou remained in a state of primary integration, while the remaining five prefecture-level cities were close to the state of integration. Therefore, the URID level of the HBUA was still low. As for the URID in China, it was lower than that of the HBUA [27]. Overall, the URID level of urban agglomeration was superior compared to small cities. Promoting the URID level of urban agglomeration and leading the URID level of small cities has been identified as a new task in China, which was indicated in the “Key Tasks for New-type Urbanization and Urban-rural Integration Development in 2022” [25].

The URID level of the HBUA showed a significant feature of spatial aggregation. This result validated Hypothesis 3. The three high-clustering areas of the URID level since 2015 were Hangzhou, Jiaxing, and Ningbo, which was a marked characteristic in the HBUA. The pattern of URID further verified the planning that was released by Zhejiang Province in 2003 [53,54], which was consistent with the result of the previous study [52]. The formulation of reasonable and effective planning and policy systems was of great significance to the overall development of urban agglomerations.

Land-use transition accelerated the spatial development and utilization between urban and rural areas, which affected the process of URID [29]. The land-use change in the HBUA from 2000 to 2020 showed a typical characteristic of the transition between cultivated land and construction land; other land-use types (i.e., woodland, waters, grassland, and unused land) were in stable states. This feature of land-use change indicated to some extent that the economic development and urbanization in the HBUA were promoted without sacrificing the environment. However, the farmland loss and the subsequent issue of food security were a concern, which was confirmed in another study of this region [73]. This phenomenon was obvious in the urbanization of China, and the “structure–function” transformation was indicated as a concern in the protection of cultivated land [74].

### 5.2. Driving Mechanism of Urban-Rural Integration Development Level Change

Urban agglomeration, as an important development mode in the process of urbanization, is a complex system with diverse interactions among the subsystems involving economy, society, and ecology [75]. These interactions are driven by human needs and can also change the quality of life. Therefore, a novel and people-oriented urbanization strategy was implemented in China [25]. All these developmental activities were based on land resources and constrained by land capacity. This study confirmed that the main driving factor of the URID level was different at the different stages of development. This result validated Hypothesis 4. The spatial integration had the most important impact on the URID level in 2000 and 2005, which could explain over 70% of URIDI. Convenient transportation is critical to a region and has been shown to reduce the time and cost to individuals and for goods [35]. Therefore, in the early stage of our URID analysis (i.e., the years 2000 and 2005), improving the spatial integration between urban and rural areas could not be ignored, as it could build a foundation for the development of economic and social integration. The social, economic, ecological, and spatial integrations were simultaneous in 2010, which could explain over 60% or even more than 90% of the URIDI. The economic and social integrations impacted more on the URIDI in 2015, which could explain over 65% of the URIDI. Population integration played the most important role in the URID process of the HBUA in 2020, which could explain over 80% of the URIDI. Migrant workers from rural areas impacted the economic, social, and cultural conditions of urban areas [34], which could also have related to the phenomenon of “village hollowing” in China [76]. Furthermore, the high-level talent flow between the areas could significantly improve the regional development, while an uneven flow would likely aggravate the existing disparity between urban and rural areas [77]. Since the “Development Plan for a High-tech Industrial Belt Around HBUA” was released in 2020 by the government of Zhejiang Province [55], population integration could play a more vital role in the URID in the future. Furthermore, reclamation activities on the coast of Hangzhou Bay have been conducted for decades, which has been harmful to the health of the coastal wetland ecosystem [78,79]. Research demonstrated that the urbanization in Hangzhou Bay has damaged the ecosystem services [80]. The protection of the fragile coastal wetland ecosystem should be a concern during the process of URID. Moreover, there was a strong synergy between the driving factors.

In summary, the evolution of URID in the HBUA included spatial integration at the early stages (from 2000 to 2005), comprehensive integration (including social, economic, ecological, and spatial integrations) at the middle stage (i.e., 2010), economic and social integrations in 2015, and primarily population integration in 2020.

### 5.3. Implication and Limitation

The URID is a new way to balance the gaps between urban and rural areas; this includes the interaction and integration of population, space, economy, society, and ecology (Figure 1). First, we must balance the population flow between urban and rural areas. The “two-entry, two-return” mechanism has been promoted in Zhejiang Province, which refers to technology entering the countryside, capital entering the countryside, youth returning to the countryside, and rural elites returning to the countryside [81]. This mechanism could promote rural revitalization. The second part is to further improve infrastructure in the HBUA, such as the infrastructure pipeline network, the living environment, and the urban renewal of old communities. In addition, actively building a large scientific and technological innovation corridor and a modern metropolitan area are also key tasks in the HBUA. The third is to achieve a diverse social integration between urban and rural areas, such as fully mobilizing the leading role of central cities (i.e., Hangzhou, Ningbo, and Jiaxing) and comprehensively promoting the construction of beautiful towns, traditional villages, small cities, and future communities. Public service facilities such as education, medical care, and information platforms should be synchronized. The fourth is to optimize the industrial structure and facilitate economic integration. It should cultivate and develop new industries and new formats. Innovating the path to realize the value of ecological products is a novel approach to the economic development of rural areas. Fifthly, urban-rural ecological integration is to promote the improvement of urban and rural living environments and the efficient use of resources when maintaining the ecological health of the region, so as to realize the equal symbiosis of urban and rural regional systems.

This study quantitatively evaluated URID levels according to the URIDI and analyzed the spatiotemporal pattern of the URIDI as well as the driving mechanisms of URID changes. Limitations were identified during the research. First, the indicators were selected based on the population–space–economy–society–ecology framework. Restricted by basic data, many other indicators were not selected in the index system. Second, only the years 2000, 2005, 2010, 2015, and 2020 were selected. The inflection points of the URID level between 2010 and 2015 could be pinpointed. Therefore, further research should investigate the changes in the URID levels and the driving mechanisms over a longer time period based on more detailed data (i.e., time-series data) in an effort to provide more comprehensive results.

## 6. Conclusions

This study constructed a population–space–economy–society–ecology framework to measure URID levels and further explore the driving mechanisms of URID changes. The results showed that the land-use change in the HBUA from 2000 to 2020 showed a typical characteristic of the transition between cultivated land and construction land, and other land-use types (i.e., woodland, waters, grassland, and unused land) were stable. The URID level in the HBUA improved from 2000 (the URIDI value is 0.294) to 2020 (the URIDI value is 0.563), and the year 2005 could have been the inflection point for URID. The URID level of the HBUA showed a significant feature of spatial aggregation with a high value. The three high-clustering areas of the URID levels since 2015 were Hangzhou, Jiaxing, and Ningbo. The population and spatial integrations had more important impacts on the URID level in 2000, 2005, and 2020, while economic and social integrations had more significant impacts on the URID levels in 2010 and 2015. This study provided a clear understanding of the evolution and the driving mechanisms of URID of urban agglomeration and may be used as a reference for the integration of urban and rural areas for decision makers.

## Figures and Tables

**Table 1:** The measurement indicator system of URID and the summary statistics of variables.

Target Layer	First-Level Indicators	Second-Level Indicators	Calculation or Description of the Indicators	Indicator Code	Indicator Property	Mean	SD
Urban-rural integration development	Population integration	Population interaction level	Proportion of the sum of the immigrant and the emigrant population to the total population, %	X1	+	1.984	1.083
		Population urbanization rate	Proportion of urban population to permanent population, %	X2	+	60.500	11.157
		Ratio of non-agricultural employment	Ratio of employees in the secondary and tertiary industries to employees in the primary industry, %	X3	+	9.147	9.105
		Road passenger volume	10,000 persons	X4	+	11,478.636	8532.097
	Economic integration	Economic development level	GDP per capita, CYN 10,000 per person	X5	+	6.685	4.384
		Per capita income ratio of urban and rural residents	Per capita disposable income of urban households/per capita disposable income of rural households	X6	+	1.957	0.214
		Dual contrast coefficient	(output value of primary industry/employees of primary industry)/(output value of secondary and tertiary industries/employees of secondary and tertiary industries)	X7	+	0.458	0.260
		Wealth status	Savings deposit of urban and rural household at the year-end/number of permanent residents, CYN 100 million per person	X8	+	4.883	3.568
	Social integration	Endowment insurance coverage rate	Number of persons in the urban and rural old-age security/number of permanent residents, %	X9	+	30.594	19.700
		Educational situation	Number of students in secondary school and above/number of permanent residents, %	X10	+	7.249	1.677
		Medical condition	Number of beds in health institutions per 10,000 people	X11	+	40.531	14.713
		Cultural construction	Number of books in public libraries per capita, volumes per person	X12	+	1.003	0.690
	Spatial integration	Land development intensity	Proportion of construction land area, %	X13	+	11.733	7.414
		Urban land expansion	Crop sewn area/construction land area	X14	+	17.894	25.209
		Traffic network density	Total length of highways/total land area, km/km^2^	X15	+	1.006	0.455
		Number of private cars owned	Civilian car ownership/number of permanent residents, vehicles per 10,000 people	X16	+	1289.364	1150.207
	Ecological integration	Urban and rural water environment status	Industrial wastewater discharge volume, 10,000 tons	X17	−	19,577.301	20,259.963
		Urban and rural pollution control capability	Comprehensive utilization rate of industrial solid waste, %	X18	+	93.804	7.631
		Urban and rural contamination status	Agricultural chemical fertilizer application amount, 10,000 tons	X19	−	7.747	4.066
		Urban and rural greening level	Greening coverage rate of built-up areas, %	X20	+	37.249	6.486

**Table 2:** The critical *p*-values and z-scores for different confidence levels.

z-Score	*p*-Value	Confidence Level
<−1.65 or >+1.65	<0.10	90%
<−1.96 or >+1.96	<0.05	95%
<−2.58 or >+2.58	<0.01	99%

**Table 3:** Types of interaction between two variables.

Graphical Representation	Description	Interaction Type	
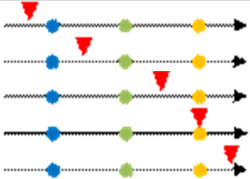	Q(X1∩X2) < Min(Q(X1), Q(X2))	Weaken, nonlinear ^1^	
	Min(Q(X1), Q(X2)) < Q(X1∩X2) < Max(Q(X1), Q(X2))	Weaken, uni- ^2^	
	Q(X1∩X2) > Max(Q(X1), Q(X2))	Enhance, bi- ^3^	
	Q(X1∩X2) = Q(X1) + Q(X2)	Independent ^4^	
	Q(X1∩X2) > Q(X1) + Q(X2)	Enhance, nonlinear ^5^	

● Min(Q(X1), Q(X2)), ● Max(Q(X1)), Q(X2)), ● Q(X1) + Q(X2), ▼ Q(X1∩X2). Q(X1) is the Q value of variable X1; Q(X2) is the Q value of variable X2; Q(X1∩X2) is the Q value of the interaction between variables X1 and X2. ^1^ Impacts of single variables are nonlinearly weakened by the interaction of two variables. ^2^ Impacts of single variables are uni-variably weakened by the interaction. ^3^ Impact of single variables are bi-variably enhanced by the interaction. ^4^ Impacts of variables are independent. ^5^ Impacts of variables are nonlinearly enhanced.

## Data Availability

Not applicable.
